# A systematic review of the long-term efficacy of low-intensity shockwave therapy for vasculogenic erectile dysfunction

**DOI:** 10.1007/s11255-019-02127-z

**Published:** 2019-03-22

**Authors:** Oliver Brunckhorst, Lauren Wells, Fiona Teeling, Gordon Muir, Asif Muneer, Kamran Ahmed

**Affiliations:** 1 MRC Centre for Transplantation, Guy’s Hospital Campus, King’s College London, King’s Health Partners, London, SE1 9RT UK; 2 Department of Urology, University College Hospital, University College London Hospitals NHS Foundation Trust, London, UK; 3 Department of Urology, King’s College Hospital, London, UK

**Keywords:** Erectile dysfunction, Vasculogenic impotence, Extracorporeal shockwave therapy

## Abstract

**Purpose:**

To look at the evidence base for LISWT as a treatment modality for vasculogenic erectile dysfunction, focusing on the long-term outcomes at over 6 months following treatment.

**Methods:**

A systematic literature search was conducted utilising MEDLINE and Scopus databases from 2010 to September 2018 by two independent reviewers. Outcome measures extracted for long-term efficacy included International Index of Erectile Function scores and Erection Hardness Scores. Subgroup analysis for LISWT effectiveness included age, PDE5i responsiveness, presence of vascular co-morbidities and smoking status.

**Results:**

The search identified eleven studies, representing a total of 799 patients. Nine studies found a significant improvement in erectile function after LISWT at 6-month follow-up (median IIEF-EF improvement in 5.3 at 6 months). However, of five studies assessing erectile function at 12 months; two identified a plateauing of results, with three a deterioration (IIEF-EF score changes of − 2 to 0.1 from 6 months). Erectile function did, however, remain above baseline results in all of these studies. Subgroup analysis revealed increasing age to reduce the response to LISWT treatment. Whilst ED severity, PDE5i responsiveness and co-morbidities potentially influence effectiveness, results are still inconsistent.

**Conclusions:**

LISWT may be a safe and acceptable potential ED treatment with demonstrated benefits at 6 months. There is some question regarding efficacy deterioration beyond this, but there is still a demonstrated benefit seen even at 12 months post treatment. However, quality of evidence remains low with larger multiinstitutional studies required, standardising confounders such as shockwave administration and oral medication use.

**Electronic supplementary material:**

The online version of this article (10.1007/s11255-019-02127-z) contains supplementary material, which is available to authorized users.

## Introduction

Atherosclerosis of penile arteries and endothelial dysfunction, known as vasculogenic erectile dysfunction (ED), is the cause of ED in 40% of men over the age of fifty [1]. There is currently no known long-lasting or curative treatment for vasculogenic ED [2]. At present both AUA and EAU guidelines for the treatment of vasculogenic ED recommend initial lifestyle changes to address modifiable risk factors, followed by oral phosphodiesterase 5 inhibitors (PDE5is) as first line medical management. However, only 80% of patients respond to PDE5is as its mechanism of action requires both intact nerves and a basic level of endothelial function [3]. PDE5is are contraindicated in patients using nitrate therapy, so a significant proportion of patients with Vascular ED are forced into second and third line treatment options [4]. Alternative treatment options to PDE5is include vacuum erection devices which are simple to use but have variable patient satisfaction rates [5], intracavernosal injections and topical prostaglandin E1 analogues (Alprostadil). Patients unresponsive to first and second line treatments may progress to surgical management with penile prosthesis [4].

The precise mechanism of action of low-intensity shockwave therapy (LISWT) is not fully understood; however, it is believed that the compression and subsequent negative pressures created from the shockwave energy, the so-called cavitation phenomenon, is an important factor [6]. These tensile forces lead to shear stress on cell membranes which have been shown to have the potential to treat the underlying cause of vascular ED by prompting increased expression of vascular endothelial growth factor (VEGF) [7], recruitment of perivascular stem cells and recruitment of endothelial progenitor cells [8], resulting in neovascularisation of penile arteries. Furthermore, shockwaves may also improve nerve regeneration as seen in animal studies, due to a hypothesised increasing in the ability of injured axons to repair and Schwann cell proliferation [9] which may be useful in ED caused by neurovascular aetiologies. LISWT is unlike any of the currently offered treatment options as it could provide men with a natural erection by treating the underlying pathophysiology, rather than treating the symptoms.

However, the evidence for its use is still debated at present, lacking Food and Drug Administration (FDA) approval for ED, and is still considered experimental by organisations such as the Sexual Medicine Society of North America. Additionally, there has so far largely been a focus on the short-term efficacy of LISWT as a treatment mobility. This review of the literature therefore aimed to:

1. Assess the current evidence base focusing on the long-term outcomes at over 6 months of using LISWT as a treatment modality for vascular ED.
2. Identify if any cohorts of patients with demonstrated improved long-term efficacy of treatment after LISWT

## Materials and methods

This review was performed following guidelines defined in the Preferred Reporting Items for Systematic Reviews and Meta-Analyses (PRISMA) statement [10]. The review was prospectively registered, PROSPERO registration number: CRD42018112789.

### Study eligibility criteria

Original research articles utilising LISWT to treat vasculogenic ED, with a minimum follow-up period of 6 months for their cohorts, were included. Study types included were randomised controlled trials (RCTs) as well as prospective and retrospective single arm experimental studies. Exclusion criteria were articles not utilising either International Index of Erectile Function-Erectile Function Domain (IIEF-EF) or Erection Hardness Score (EHS) as an outcome parameter and studies published before 2010 (the first trial for LISWT in vasculogenic ED was conducted in 2010 [2]). Furthermore, animal studies, case studies, reviews, studies using LISWT for non-vasculogenic ED and studies unavailable in the English language were all excluded from the review.

### Information sources and search

Electronic databases, MEDLINE (via PubMed) and Scopus, were systematically searched for research articles from January 2010 to September 2018. A combination of MeSH terms and key terms was used (‘Low-Intensity Shockwave Therapy’ OR ‘Pulsed Ultrasound’ OR ‘Low Intensity Ultrasound’ OR ‘Shockwave’ OR ‘Shock wave’) AND (‘Erectile Dysfunction’ OR ‘ED’ OR ‘Sexual Dysfunction’). In addition, a thorough reference review of identified articles was conducted, to ensure that all relevant articles were included. The grey literature was searched via abstracts on Scopus and ongoing clinical trials in Cochrane Library and ClinicalTrials.gov, with authors contacted for any available preliminary data.

### Study selection

The search was conducted independently by two reviewers (OB and LW) to identify potentially relevant articles. Title and abstract screening was conducted, with full-text articles subsequently screened along inclusion criteria for inclusion into qualitative analysis. Discrepancies between reviewers were discussed until 100% agreement was achieved.

### Data collection and data items

Data extraction was independently conducted by two reviewers (OB and LW). Specific data were extracted from all studies such as study type, number of participants, participant demographics and LISWT treatment regimen. The primary outcome measure extracted for clinical efficacy included erectile function measures such as IIEF-EF or EHS scores after LISWT at long-term follow-up of over 6 months. This included both raw questionnaire score improvements, percentage improvements and also study defined success rates as per score improvements. Additionally, subgroup analysis of LISWT effectiveness was conducted via assessment of population cohorts including age, PDE5i responsiveness, presence of vascular co-morbidities and smoking status.

### Risk of bias in individual studies and across studies

The internal validity of each individual article was assessed using the Cochrane Risk of bias tool and with further qualitative analysis for randomised controlled trials and non-randomised studies, respectively [11]. Non-randomised studies were assessed qualitatively by authors critically appraising the methodology, as per definitions in Online Resource 1. Bias across studies was assessed via the GRADE tool in order to provide a recommendation from our review for each individual outcome measure [12].

## Results

### Study selection

**Fig. 1:**
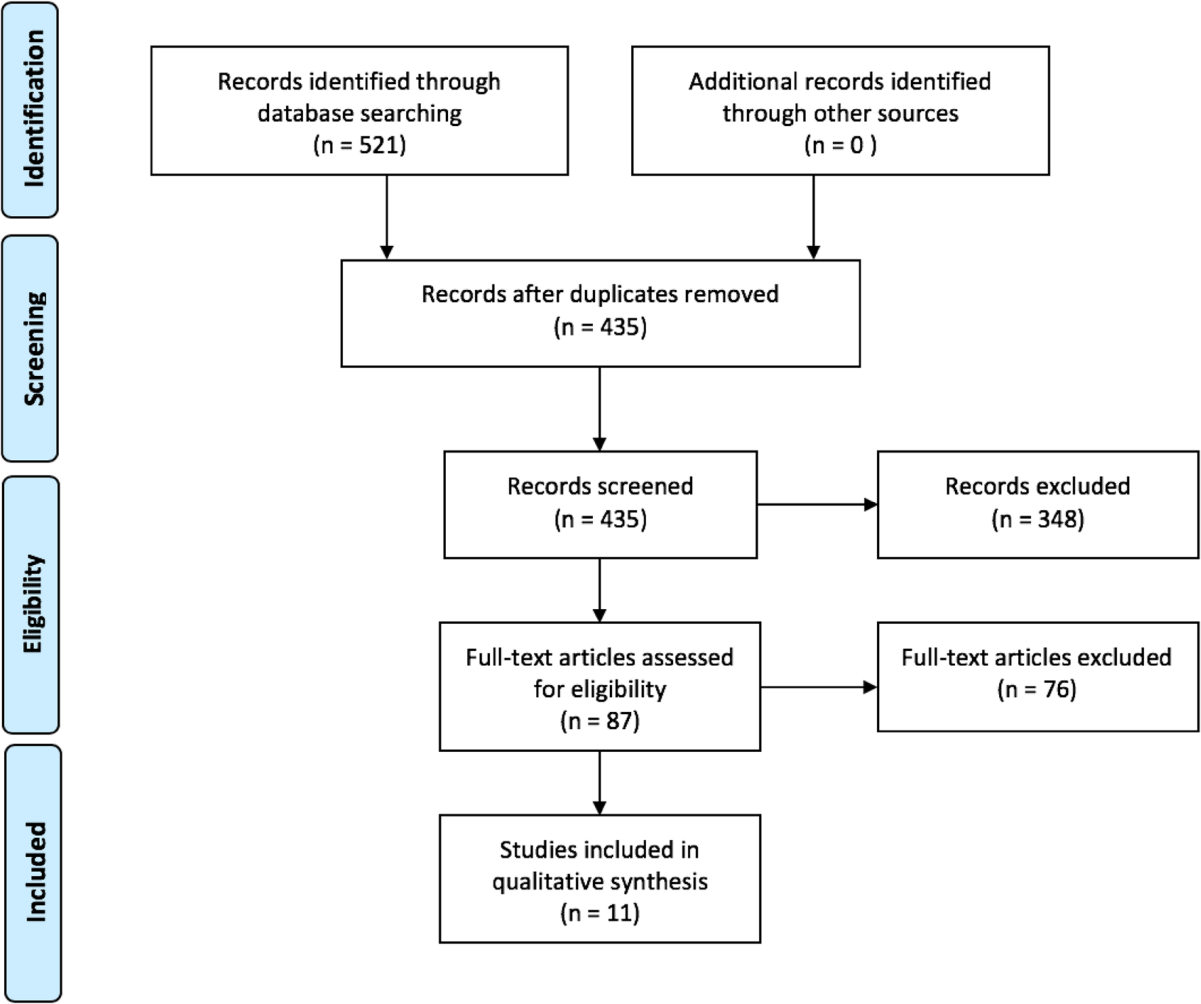
PRISMA flow chart for article selection

A total of 521 articles were identified through the literature search. Duplicate removal and initial screening excluded 434 articles. Of the 87 full-text articles assessed for eligibility, a final eleven articles were included in the review (Fig. 1).

### Study characteristics and result synthesis

Of the eleven articles, five were RCTs and six were non-randomised (Table 1). Three of the RCTs were placebo-controlled; the others compared two different treatment groups. The total number of patients investigated across all studies was 799 patients.

**Table 1:** Overview of Studies Included

Study	Follow-up duration (Months)	N. of participants	Study design	Baseline IIEF Score for inclusion	Stable heterosexual relationship > 3 m	PDE5i responsive?	Shockwave exposure	Shockwave administration location	Shockwave delivery	Sig. improvement using LISWT at > 6 months?
Bechara 2016 [17]	12	40	Prospective Single-Arm Trial	< 26	Not stated	N	F: Once per week D: 4 weeks	Each Corpora cavernosum and perineum (each crus)	*TSWPS*: 3,600, *SWD*: 900 (per corpora), 900 (per crus) *EFD*: 0.09 mJ/mm^2^, *F*: not given	Y
Fojecki 2018 [20]	12	126	Double-Blinded Sham-Controlled Randomised Trial	< 25	Y	Not stated	F: Once per week D: 5 weeks (just group A) 4-week break	Each corpora cavernosum and perineum (each crus)	*TSWPS*: 600 *SWD*: 300 (2 corpora), 150 (per crus) *EFD*: 0.09 mJ/mm^2^, *F*: 5 Hz	N
Hisasue 2016 [16]	6	56	Prospective Single-Arm Trial	Not stated (EHS of 1 or 2)	Not stated	Not stated	F: Twice per week D: 3 weeks, 3-week break and 3 weeks	Three sites on one side of penile shaft, two on crura	*TSWPS*: 1500, *SWD*: 300 (3 penile sites and per crus) *EFD*: 0.09 mJ/mm^2^, *F*: 120/min	Y
Kalyvianakis 2018 [13]	6	42	Randomised, 2-parallel-arm, open-label study	< 26	Not stated	Not stated	F: Once or Twice per week dependent on Group D: 12 weeks, (Phase 1: 6 weeks, Phase 2: 6 weeks)	Back and forth movement of probe from glans to pubis on each side	*TSWPS*: 5000 *SWD*: 1000 (per corpora and 2 penile site), 500 (per crus) *EFD*: 0.05 mJ/mm^2^, *F*: 8 Hz	Y
Kalyvianakis 2017 [14]	12	46	Double-Blinded Sham-Controlled Randomised Trial	6–21	Y	Y (at least partial)	F: Twice per week D: 3 weeks, 3-week break and 3 weeks	Three locations on penile shaft, two on crura	*TSWPS*: 1500, *SWD*: 300 (3 penile sites and per crus) *EFD*: 0.09 mJ/mm^2^, *F*: 160 pulses/min	Y
Kitrey 2018 [12]	24	156	Prospective Single-Arm Trial	Not stated	Not stated	Mixed	F: Twice per week D: 3 weeks, 3-week break and 3 weeks	Five treatment points on penile shaft	*TSWPS*: 1500, *SWD*: 300 (5 penile sites) *EFD*: 0.09 mJ/mm^2^, *F*: 120 pulses/min	Y
Olsen 2015 [21]	6	105	Double-Blinded Sham-Controlled Randomised Trial	< 20	Y	Y	F: Once per week D: 5 weeks	Three positions on each corpora cavernosum individually	*TSWPS*: 3000, *SWD*: 500 (3 per corpora) *EFD*: 0.15 mJ/mm^2^, *F*: 5 Hz	N
Pelayo-Nieto 2015 [18]	6	15	Prospective Single-Arm Trial	12–16	Not stated	Not stated	F: Once per fortnight D: 8 weeks	Two points on cavernosa and each crus	*TSWPS*: 5,000, *SWD*: 900 (per corpora), 1600 (per crus) *EFD*: 0.09 mJ/mm^2^, *F*: 5 Hz	Y
Reisman 2014 [19]	6	58	Prospective Single-Arm Trial	6–25	Y	Mixed	F: Once per week D: 4 weeks	One site on each corpora cavernosum and crus	*TSWPS*: 3600, *SWD*: 900 (per corpora and crus) *EFD*: 0.09 mJ/mm^2^, *F*: not given	Y
Srini 2015 [15]	12	135	Double-Blinded Sham-Controlled Randomised Trial	< 18	Y	Y	F: Twice per week D: 3 weeks, 3-week break and 3 weeks	Five treatment points unilaterally on penile shaft	*TSWPS*: 1500, *SWD*: 300 (5 penile sites) *EFD*: 0.09 mJ/mm^2^, *F*: 120/min	Y
Vardi 2010 [2]	6	20	Prospective Single-Arm Trial	5–19	Not stated	Y	F: Twice per week D: 3 weeks, 3-week break and 3 weeks	Three points on penile shaft and each crura	*TSWPS*: 1500, *SWD*: 300 (3 penile sites and per crus) *EFD*: 0.09 mJ/mm^2^, *F*: 120/min	Y

*D* duration, *EFD* energy flux density, *F* frequency, *LISWT* low-intensity shockwave therapy, *SWD* shockwave delivery, *TSWPS* total shockwaves per session

### Long-term efficacy of LISWT

**Fig. 2:**
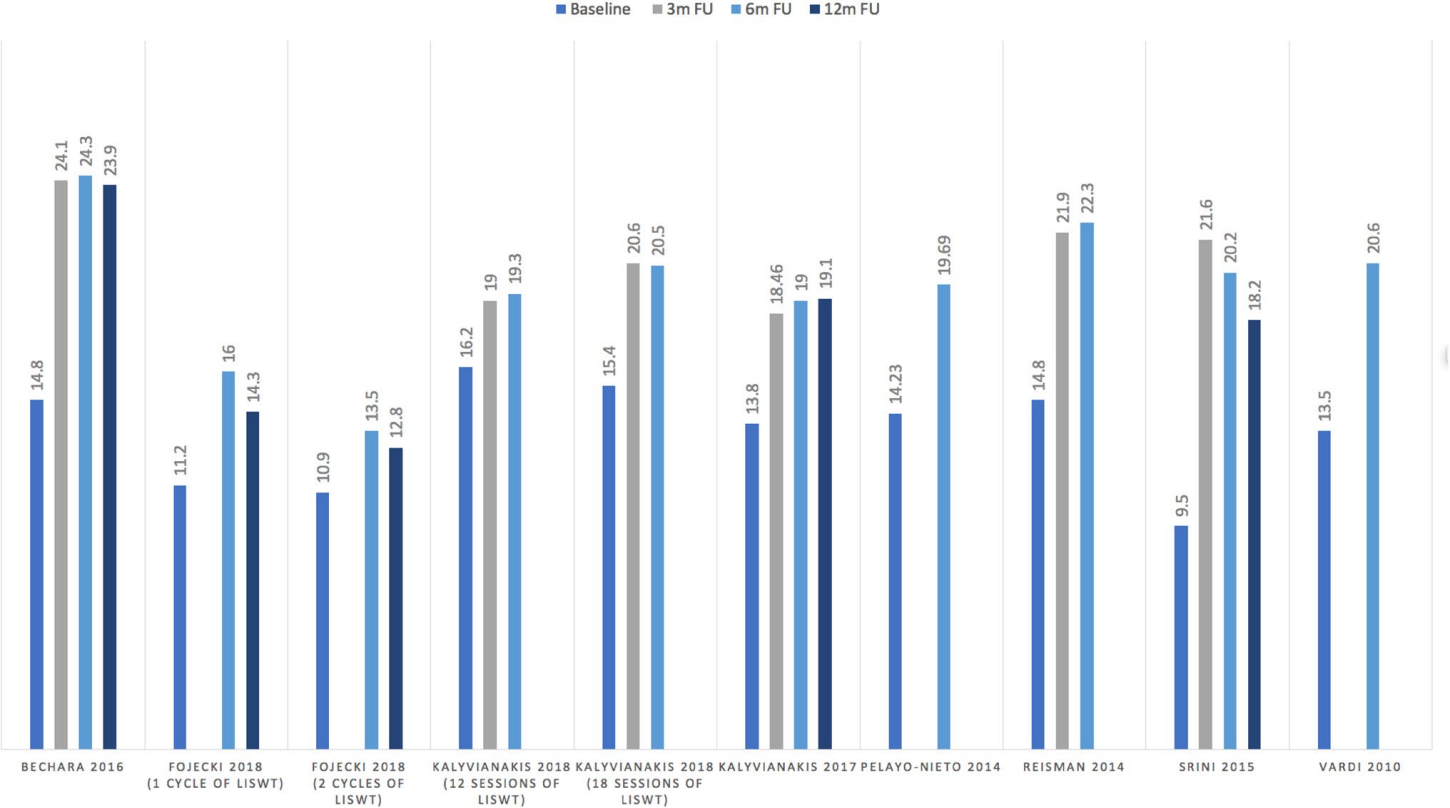
Long-term efficacy of LISWT based on IIEF-EF scores at baseline, 3, 6 and 12 months

Nine studies of the eleven studies [2, 13–20] found a statistically significant increase in erectile function utilising either IIEF or EHS scores after LISWT at over 6-month follow-up (median IIEF-EF score improvement from baseline at 6 months 5.3, range 2.6–10.7). None of these studies demonstrated a decrease in erectile function below baseline post intervention (Fig. 2). However, the results from two randomised, sham-controlled studies, Fojecki et al. and Olsen et al., did not reach the authors’ set threshold for significance at follow-ups of over 6 months [21, 22]. The effects of LISWT were followed up for 24 months in one study, 12 months in four studies and 6 months in the remaining six studies. When assessing studies with follow-ups of greater than 6 months, there appeared to be limited improvement in IIEF scores beyond this time period (change in IIEF-EF scores of between − 2 and 0.1). No studies identified an ongoing improvement at 12 months when compared to 6 months with two studies demonstrating plateauing of IIEF scores [15, 18]. Three studies showed there was a gradual diminishing effect of effectiveness of LISWT beyond 6 months; however, scores remained at above baseline erectile function in all cases [13, 16, 21]. The largest of these studies demonstrating a gradual decline was conducted by Kitrey et al. This prospective single-armed trial of 156 patients demonstrated an initial response rate of 63.5% at 1 month, decreasing to 42.9% at 12 months and declining to just 34% at 2-year follow-up.

### Effect of LISWT on specific population cohorts

Subgroup analysis demonstrated conflicting findings between studies. Whilst the majority of studies have not been powered to draw conclusions based on specific subgroups, trends in results were seen. Age, PDE5i responsiveness, presence of vascular co-morbidities and smoking status have all been proposed to impact the efficacy of LISWT treatment.

Two out of three studies assessing age specifically identified a reduced effectiveness of LISWT with increasing age [14, 17]. These studies identified that younger age was a statistically significant predictor for improved treatment responsiveness and increasing age (> 65 years) and vascular co-morbidities shortened the duration of LISWT effects in comparison to younger, healthier patients. However, in contrast the study conducted by Bechara et al. saw no statistically significant difference in age, duration of ED or co-morbidities when comparing LISWT responders to non-responders [18].

ED severity appears to have contrasting effects on efficacy. Whilst the study conducted by Bechara et al. identified that patients with severe ED responded better to LISWT with the greatest IIEF-EF point increase, Kitrey and colleagues found that the duration of treatment effect was reduced in patients with severe ED at 24-month follow-up [13, 18]. Additionally, when considering duration of ED symptoms, Reisman et al. identified a negative effect on LISWT responsiveness with increasing duration of symptoms, finding the average ED duration to be 3.5 years longer in LISWT non-responders than responders [20]. PDE5i response was seen to be important in a single study where PDE5i responders were statistically more likely to receive benefit from LISWT, with longer duration of efficacy in this cohort [14].

Two studies assessed the effect of vascular risk factors on LISWT effectiveness. In a comparison of patients with at least one vascular co-morbidity (cardiovascular disease, hypertension, high cholesterol), to those with none, Reisman et al. identified lower success rates in those with co-morbidities (76.2% vs 93.7%, respectively) [20]. Smoking status was seen to negatively impact the success of LISWT in one study, with patients possessing a smoking index of less than 20 having a statistically significant chance of improving erectile function (91% vs. 50%) [19]. Finally, the presence of diabetes demonstrated mixed results. Whilst one study demonstrated success rates which were 25% lower in diabetic patients, with shorter duration of treatment effects [20], Pelayo-Nieto et al. contrastingly identified that diabetic patients demonstrated an improved clinical response to LISWT (62% vs 47%) [19].

### Quality assessment of articles

There are still limited number of studies assessing long-term follow-up after LISWT with predominantly non-randomised trials present. Risk of bias assessment of randomised trials (Online Resource 2) showed the largest concern to be regarding selection bias, introduced by attrition of study participants. Whilst higher attrition rates are expected due to the longer follow-up in our selected articles, unusually high dropout rates in some studies such as Srini and Fojecki et al. (over 20%) which was identified to be more heavily weighted towards the placebo or the 5-week treatment group [16, 21]. This could skew results towards those receiving the treatment, generating false-positive results. Additionally, the five randomised trials demonstrated small numbers of total participants, with all being single centre trials. There are finally concerns regarding the sham or double blinded nature of the trials. It is difficult to ensure true blinding in these circumstances with many of the trials identifying no benefit at all from sham treatment which is unexpected as some placebo effect is expected.

PDE5i use prior and during treatment is currently variable. Whilst the majority of studies included a 4-week ‘washout period’ without PDE5i use, this was not consistent across all studies, with additional variation surrounding ongoing PDE5i use and timing of restarting this. Four studies allowing participants to resume PDE5i use from 1-month post-LISWT [2, 17, 20, 21], and one study keeping patients on treatment throughout entire treatment duration [18]. This is important as this produces as a confounder which may explain differences in long-term efficacy post-LISWT. Finally, an extremely large variation in administration protocols was identified. Individual studies varied significantly in terms of shockwaves delivered per session, time between sessions and even sites of administration as demonstrated in Table 1. This presents a limitation towards external validity of the studies in view of effect sizes for long-term erectile function post-LISWT.

## Comment

This systematic review assesses the long-term effect of LISWT at over 6 months on vasculogenic ED patients. Out of the eleven papers identified, nine demonstrated a statistically significant improvement in erectile function at 6-month follow-up. However, these studies show that beyond 6 months there is no ongoing improvement in erectile function seen. Three out of five studies demonstrated a gradual decline in erectile function with two showing a plateauing of results. However, it is important to note that IIEF-EF scores in all studies remained significantly above baseline functional scores, demonstrating benefit even at 12-month post-treatment. This is likely secondary to the ongoing underlying vascular progression of disease, with LISWT having no impact on comorbidities or progressive atherosclerotic disease affecting the cavernosal tissue [13]. Assessment of the overall quality of the evidence for long erectile function improvement via the GRADE protocol demonstrates that current recommendation for use remains low at present (Online Resource 3). This is due to the predominantly non-randomised evidence base currently present with trials presenting small patient numbers, single institutions and methodological concerns regarding the double-blind sham trials.

Subgroup analysis assessing individual cohorts of patients for LISWT effectiveness yielded varying results. Two studies suggest that younger patients may be more likely to benefit from prolonged benefits in erectile function. This is hypothesised to be secondary to less structural cavernosal and ultra-structural damage present, with greater VEGF receptor activity resulting in a greater biological response from treatment [14, 17]. This has led to suggestions that LISWT may have a role in early intervention, or even prophylactic treatment in high-risk patients, thereby preventing irreversible vascular changes [14, 23]. However, at present the objective evidence for this is non-existent. Similarly, it would be expected that patients with severe ED would see a reduced effectiveness of treatment secondary to increased ultra-structural damage. Whilst previous findings in short-term follow-up have supported this [24], our review has identified conflicting evidence in the long term, with no clear relationship of ED severity or duration to clinical efficacy.

The evidence assessing cardiovascular co-morbidities and risk factors on treatment effect in the long term is limited. There is contrasting evidence assessing diabetic patients with further review certainly needed. Furthermore, whilst smoking and presence of other cardiovascular risk factors appear to negatively impact the efficacy of treatment, these results are restricted to a single study only in the long term [20]. Similarly, whilst PDE5i responders and naïve patients have previously been identified as positive predictive markers for treatment success in short term studies [24], this cannot be determined for long-term studies yet. Only a single study demonstrated improved efficacy; however, this was not statistically significant. Therefore, at present there is no concrete evidence for any subgroup identified as a predictive marker for long-term successful treatment.

This is the first review, to our knowledge, to specifically assess the long-term efficacy of LISWT for vasculogenic ED. It provides important evidence to demonstrate that there appears to be a lasting effect of erectile function improvement at 6 months for patients which either plateau or may gradually deteriorate towards 12 months post-treatment. This is clinically important, providing urologists evidence for treatment, but additionally offers evidence for frank discussion regarding expectations of erectile function beyond 6-month post-treatment. However, as previously mentioned due to varying study methodologies this review highlights to researchers further areas of research to increase the evidence base surrounding LISWT use.

Whilst there is clinically relevant data currently available, there are several concerns regarding current methodology of identified trials which require standardisation in future studies. It is clear larger studies which are multi-institutional and multi-national are required in the first instance to increase external validity of results. Further to this, PDE5is use prior and post-treatment is extremely variable in the literature. Whilst a washout period and limited PDE5i use may improve the results obtained, it could be argued that future research should focus on more real-life applicability by maintenance of medical therapy concurrently to LISWT. Additionally, future research must standardise administration of LISWT, in particular with regard to the device utilised, treatment delivery in terms of sessions and duration as well as location of administration with current positioning widely varied in the studies identified. In terms of outcome measures utilised, there is a need for greater objective parameters through penile haemodynamic studies concurrently to subjective measures such as IIEF-EF and EHS scores, which can be heavily influenced by other factors such as sexual partners, lifestyle, life events, psychology and comorbidities [13]. Finally, this review has identified a need for longer follow-up at 12 months and beyond to assess the ongoing longevity of treatment efficacy.

As is the case with any systematic review, this review has its limitations. These are largely arising from the data set available with limitations in the number of trials which are available with few randomised studies identified assessing long-term outcomes specifically. Additionally, several methodological concerns and variations as previously mentioned are present. This is particularly true when considering treatment administration and concurrent therapy, meaning that generalisability of results must be considered, and hence, standardised recommendations could not be made. When this variability in methodology was combined with the lack of randomised trials reporting standardised outcome measures such as IIEF-EF and the large heterogeneity of results seen when statistical pooling of randomised studies was attempted, meant that any meaningful statistical assessment of data via a meta-analysis was not feasible. Finally, there is always the possibility of missed studies which could affect current recommendation; however, the risk of this was minimised via a comprehensive search strategy and searching both grey and current literature.

## Conclusions

This systematic review identifies that LISWT offers a treatment modality which improves erectile function, with results lasting to over 6 months. There appears to be some limitation of ongoing benefit beyond this at 12 months with either plateauing or even reduction in functional outcomes at this time. Increasing age appears to reduce responsiveness to LISWT treatment in long-term follow-up studies. Furthermore, ED severity, PDE5i responsiveness and co-morbidities may also influence its effectiveness; however, results are inconsistent at present. Whilst LISWT may be a safe and acceptable long-term ED treatment modality, it is clear further investigation is still needed through larger and more standardised trials to improve its evidence base.

## Electronic supplementary material

Below is the link to the electronic supplementary material.

Supplementary material 1 (PDF 41 KB)

Supplementary material 2 (PDF 63 KB)

Supplementary material 3 (PDF 52 KB)
