# Supplementary material for: A systematic review of the long-term efficacy of low-intensity shockwave therapy for vasculogenic erectile dysfunction
Source: Int Urol Nephrol. 2019 Mar 22;51(5):773–81. doi: 10.1007/s11255-019-02127-z (PMC6499893; doi:10.1007/s11255-019-02127-z)
Supplement: Supplementary file 1 — Supplementary material 1 (PDF 41 KB) [file 11255_2019_2127_MOESM1_ESM.pdf]

Online Resource 1: Types of Bias to assessed per article (developed from Cochrane)		
**Type of Bias**	**Description**	**Relevant Domains in the Risk of Bias Tool**
**Selection Bias**	Systematic differences between the baseline characteristics of groups compared	▪ Sequence Generation<br>▪ Allocation Concealment
**Performance Bias**	Systematic differences between groups in the care provided/exposure other than the intervention being measured	▪ Blinding of participants and personnel<br>▪ Other potential threats to validity
**Detection Bias**	Systematic differences between groups in how outcomes are determined	▪ Blinding of outcome assessment<br>▪ Other potential threats to validity
**Attrition Bias**	Systematic differences between groups in withdrawal from a study	▪ Incomplete outcome data
**Reporting Bias**	Systematic differences in reported and unreported findings	▪ Selective outcome reporting
