# Supplementary material for: A systematic review of the long-term efficacy of low-intensity shockwave therapy for vasculogenic erectile dysfunction
Source: Int Urol Nephrol. 2019 Mar 22;51(5):773–81. doi: 10.1007/s11255-019-02127-z (PMC6499893; doi:10.1007/s11255-019-02127-z)
Supplement: Supplementary file 2 — Supplementary material 2 (PDF 63 KB) [file 11255_2019_2127_MOESM2_ESM.pdf]

Online Resource 2 : GRADE – Summary of Findings Table

Outcome Measure	Estimate of Effect based on Study Design	Final Rating of Quality	Overall Rating for Quality of Evidence	Factors Affecting Recommendation	Direction and Strength of Overall Recommendation
What is the Long-Term Efficacy of LISWT (≥6m)?	Vardi 2010 (LOW)	MODERATE<br>+1, used objective measurements of penile haemodynamics to compare against patient reported outcomes	LOW (++)	Quality of Evidence:<br>The main body of evidence is from non-randomised studies and low-quality RCTs.<br><br>Balance of Desirable/Undesirable Outcomes:<br>6 studies supporting LISWT as an effective treatment and 2 studies finding no significance.<br><br>Values and Preferences:<br>Minimal side effects and well tolerated by all patients	Weak recommendation for using LISWT. Requirement for additional high quality evidence.
	Bechara 2016 (LOW)	LOW			
	Srini 2016 (HIGH)	LOW<br>-2, due to high risk of selection bias due to high attrition rate			
	Hisasue 2016 (LOW)	LOW			
	Reisman 2014 (LOW)	LOW			
	Pelayo-Nieto 2015 (LOW)	LOW			
	Olsen 2015 (HIGH)	MODERATE<br>-1, possible selective reporting of outcomes due to no published raw data, no explanation of discrepancy between findings on IIEF-EF and EHS			
	Fojecki 2018 (HIGH)	LOW<br>-2, high attrition rate within participants, and significant differences between participants in treatment groups in terms of co-morbidities			
	Fojecki 2018 (HIGH)	VERY LOW<br>-2, high attrition rate within participants, and significant differences between participants in treatment groups in terms of co-morbidities, results did not reach clinical significance for this outcome measure			
