# Supplementary material for: A systematic review of the long-term efficacy of low-intensity shockwave therapy for vasculogenic erectile dysfunction
Source: Int Urol Nephrol. 2019 Mar 22;51(5):773–81. doi: 10.1007/s11255-019-02127-z (PMC6499893; doi:10.1007/s11255-019-02127-z)
Supplement: Supplementary file 3 — Supplementary material 3 (PDF 52 KB) [file 11255_2019_2127_MOESM3_ESM.pdf]

Online Resource 3: Risk of Bias Summary Table for Included Randomised Studies

	Srini 2015	Olsen 2015	Kalyvianakis 2018	Kalyvianakis 2017	Fojecki 2018
Random sequence generation (selection bias)	+	+	+	+	+
Allocation concealment (selection bias)	+	+	+	+	-
Blinding of participants and personnel (performance bias)	?	+	?	+	+
Blinding of outcome assessment (detection bias)	?	?	+	+	-
Incomplete outcome data (attrition bias)	-	+	+	+	-
Selective reporting (reporting bias)	?	-	+	+	+
Other bias	?	+	+	+	-
